# Characterization of Recombinant Chimpanzee Adenovirus C68 Low and High-Density Particles: Impact on Determination of Viral Particle Titer

**DOI:** 10.3389/fbioe.2021.753480

**Published:** 2021-11-04

**Authors:** Elise K. Mullins, Thomas W. Powers, Jim Zobel, Kory M. Clawson, Lauren F. Barnes, Benjamin E. Draper, Qin Zou, Joseph J. Binder, Stanley Dai, Kun Zhang, Olga Friese, Herbert A. Runnels, Martin F. Jarrold, Lawrence C. Thompson

**Affiliations:** ^1^ Analytical Research and Development, Biotherapeutic Pharmaceutical Sciences, Pfizer Inc., Chesterfield, MO, United States; ^2^ Chemistry Department, Indiana University, Bloomington, IN, United States; ^3^ Megadalton Solutions, Bloomington, IN, United States; ^4^ Cancer Vaccines and Immunotherapies, Pfizer Inc., San Diego, CA, United States; ^5^ Nektar Therapeutics, San Francisco, CA, United States

**Keywords:** non-human primate, adenovirus, AEX-HPLC, analytical ultracentrifugation, differential centrifugation sedimentation, charge detection mass spectrometry, low density viral particles, viral particle titer

## Abstract

We observed differential infectivity and product yield between two recombinant chimpanzee adenovirus C68 constructs whose primary difference was genome length. To determine a possible reason for this outcome, we characterized the proportion and composition of the empty and packaged capsids. Both analytical ultracentrifugation (AUC) and differential centrifugation sedimentation (DCS, a rapid and quantitative method for measuring adenoviral packaging variants) were employed for an initial assessment of genome packaging and showed multiple species whose abundance deviated between the virus builds but not manufacturing campaigns. Identity of the packaging variants was confirmed by charge detection mass spectrometry (CDMS), the first known application of this technique to analyze adenovirus. The empty and packaged capsid populations were separated via preparative ultracentrifugation and then combined into a series of mixtures. These mixtures showed the oft-utilized denaturing A260 adenoviral particle titer method will underestimate the actual particle titer by as much as three-fold depending on the empty/full ratio. In contrast, liquid chromatography with fluorescence detection proves to be a superior viral particle titer methodology.

## Introduction

Non-human primate adenoviral vectors are attractive therapeutic vectors as they share similar beneficial characteristics of human adenovirus like robust antigen expression, but with reduced levels of neutralizing antibodies (Capone et al., 2013; Cheng et al., 2015; Zhang et al., 2017; Zhao et al., 2018). Recent successes using these constructs include modified chimpanzee adenovirus type 3 ebolavirus vaccine (cAd3-EBO) (Stanley et al., 2014; Ledgerwood et al., 2017) and replication-deficient simian adenoviral vectored vaccine ChAdOx1 nCoV-19 against SARS-CoV-2 (Folegatti et al., 2020; Ewer et al., 2021; Putter, 2021).

Fundamentally, recombinant viral vectors have a genomic composition different than native virus. For adenoviral constructs, this often means replacement of the E1 gene, possibly the E3 or other genes with the genetic payload of choice (Farina et al., 2001; Tatsis et al., 2006). These alterations as well as changes in manufacturing, including scale and/or process, can have an impact on the ordered construction of a viral particle during adenovirus maturation (Mangel and San Martín, 2014; Ahi and Mittal, 2016). The final product could contain capsids without genomes (unpackaged/empty), with complete genomes (packaged/full) or myriad intermediates in between (Vellekamp et al., 2001; Sutjipto et al., 2005).

Numerous techniques are available to provide this capsid content information (i.e., empty, partially packaged or fully packaged). The most commonly applied intact capsid approaches are dynamic light scattering (DLS) (Kondylis et al., 2019), electron microscopy (EM) (Kondylis et al., 2019), AUC (Berkowitz and Philo, 2007; Berkowitz, 2008; Yang et al., 2008) and DCS (Bondoc and Fitzpatrick, 1998; Shih et al., 2010). They can measure a range of viral particle populations from low density immature particles up to high mass aggregates. Capsid protein analysis via reversed-phase high performance liquid chromatography (RP-HPLC) can also parse out particle maturity via analysis of viral protein content and processing (Lehmberg et al., 1999; Vellekamp et al., 2001; Chelius et al., 2002; Takahashi et al., 2006). In addition to the more routine techniques, additional-state-of-the-art techniques, including charge detection mass spectrometry (CDMS) (Contino and Jarrold, 2013; Pierson et al., 2016; Draper et al., 2018; Draper and Jarrold, 2019) are available for direct mass determination of intact viral particles (Kondylis et al., 2019; Werle et al., 2021).

The current study was initiated to evaluate the performance of all of these techniques to elucidate particle content on two chimpanzee adenovirus C68 (Farina et al., 2001; Cohen et al., 2002; Xiang et al., 2002; Tatsis et al., 2006) constructs (heretofore known as AdC68 #1 and #2). AdC68 #1 and #2 each contain tumor antigens cloned in place of E1 and E3, but due to differing inserted genes, the resulting recombinant viruses contain genomes of disparate lengths. Throughout this study, we showed that DCS, AUC, and A260/A280 ratio were able to elucidate particle content while RP-HPLC and CDMS provided valuable orthogonal information. A byproduct of this investigation was the important observation that genome packaging ratios have a pronounced impact on viral particle titer accuracy via the denaturing A260 method (Maizel et al., 1968b). We demonstrate that for adenovirus products with some level of empty capsids, viral particle concentration determined by AEX-HPLC (Shabram et al., 1997; Blanche et al., 2000; Klyushnichenko et al., 2001; Kuhn et al., 2007; Whitfield et al., 2009) using native protein fluorescence detection is superior, as the technique generates an unbiased, total viral particle titer.

## Materials and Methods

### Formulation

AdC68 was formulated in A195 buffer (Evans et al., 2004): 10 mM Tris, 75 mM NaCl, 5% w/v sucrose, 0.02% (w/v) polysorbate 80, 1.0mM MgCl_2_, 0.1 mM EDTA, 0.5% (v/v) ethanol, 10 mM l-histidine, pH 7.4.

### Human Adenovirus Type 5 Reference Material (ARM)

The Human Adenovirus Type 5 Reference Material was purchased from the ATCC ARM working group (Hutchins et al., 2000; Hutchins, 2002; Berkowitz, 2008). It is a highly characterized material, containing nearly 100% packaged capsids. This allows it to function as a standard to which all other adenovirus reference materials can be quantitated against.

### Analytical Ultracentrifugation per Sedimentation Velocity

The samples were run in triplicate using a Beckman Coulter Analytical Ultracentrifuge Proteome XL/I at 5,000 RPM at 20°C. Data was collected on both the interference and absorbance at 260 nm, then 50 scans were used for analysis. The resulting data was analyzed using Sedfit (ver.15.01b) to generate c(s) size distribution plots. Sednterp (ver. 1.09) was used to determine the solvent viscosity and density.

### Differential Centrifugation Sedimentation via Disc Centrifuge

The differential centrifugation sedimentation technique utilizes a disc spun up to 24,000 rpm. A sucrose gradient was added to the disc that ranged from 8 to 24% sucrose. AdC68 samples were then injected into the disc for separation within the sucrose gradient into empty, intermediate, and full/mature species based on their density. The method reports relative percent full, empty, and intermediate species.

### Preparative Ultracentrifugation

AdC68 particles were separated by cesium chloride (CsCl) density gradient ultracentrifugation. Ultracentrifugation tubes were loaded with 5 ml of 4 M CsCl underneath 18 ml of 2.2 M CsCl. Viral particles (15.5 ml) were gently overlayed on top of the CsCl, and the tubes were spun at 25,000 rpm for 17 h at 4°C in an SW28 rotor in an Optima XPN-80 Ultracentrifuge (Beckman Coultre). Two visible bands were pulled with 3 ml syringes and transferred to dialysis cassettes with 10,000 Da molecular weight cutoff. Viral particles in the high-and-low density bands were separately dialyzed twice in A195 buffer for 4 h at 4°C before further analysis.

### Cell Culturing

Cultures of HEK293 cells (Cell biolabs, Catalog No. AD-100) were grown in a flask containing Dulbecco’s modified medium (Gibco, Catalog No. 11995) supplemented with 10% Heat Inactivated Fetal Bovine Serum (Gibco, Catalog No. 10082) and 1x Penicillin-Streptomycin (Gibco, Catalog No. 15140) in a 5% CO_2_ atmosphere at 37°C.

### Determination of the Adenoviral Infectivity by a Cell-Based Adenovirus Titer Immunoassay

This method was developed internally in Pfizer to monitor the adenovirus sample infectivity. In this assay, HEK293 cells were seeded into a 24-well flat bottom cell culture plate at 2.2E5 cells/well and then incubated in a 37°C, 5% CO2 incubator. A dilution series of adenovirus sample was prepared in cell culture medium. The dilution series was then added to the wells of the cell assay plate about 1 h after cell seeding. The assay plate was then incubated for 2 days. Following infection, the adenovirus will express the viral proteins including Hexon proteins. The cells were then fixed with cold methanol at -20°C and then stained with a polyclonal antibody against adenovirus Hexon proteins. Positive stained cells in assay wells were counted under microscope fitted with a 10x lens. The cell assay plate wells that contains 7 to 79 stained cells per view field were recorded and used to calculate the adenovirus sample infectivity, reported as IFU/ml as well as vp/IFU.

### Intact Protein RP-HPLC of Low and High-Density Fractions

The RP-HPLC method provides a capsid protein map. The samples were denatured with ACN and TFA, then separated on a Phenomenex Jupiter C4, 5µm, 2 × 150mm, 300Å analytical column. Detection is performed with UV at 214 nm to provide chromatograms that can be visually compared for peak consistency.

### CDMS of Low and High-Density Fractions

In CDMS the m/z and charge of individual ions are simultaneously measured and then multiplied to give the mass. The CDMS instrument employed here has been described in detail elsewhere (Contino and Jarrold, 2013; Draper et al., 2018; Draper and Jarrold, 2019). Briefly, ions generated by nanoelectrospray, enter the instrument though a metal capillary and pass through several differentially-pumped stages to remove the ions from the ambient gas flow. The resulting ions are accelerated and focused into a hemispherical deflection energy analyzer which transmits a narrow band of ion kinetic energies. The ions are then focused into an electrostatic linear ion trap (ELIT) which consists of two endcaps that can be switched between reflection and transmission mode. Trapped ions oscillate between the endcaps. A detection cylinder placed between the endcaps picks up the charge induced by the oscillating ions. The resulting signal is digitized and analyzed by fast Fourier transforms. The oscillation frequency is related to the ion’s m/z and the charge is obtained from the FFT magnitude.

The adenovirus samples were stored in a -80°C freezer prior to analysis. Aliquots were thawed at room temperature and prepared for analysis in a class II biosafety cabinet (NuAire LabGard ES type A2). The sample was desalted and buffer exchanged via size exclusion chromatography (Micro Bio-Spin P-6 Gel Columns, Bio-Rad) into a 200 mM ammonium acetate (Honeywell 631-31-8). Measurements were performed for thousands of ions over a span of 30–45 min and then the masses were binned (0.5 MDa bins) to give the mass spectrum.

### Stability Sample Degradation Description

Approximately 120 ml each of AdC68 #1 material and 120 ml of AdC68 #2 material were added to respective Nalgene bottles. A 10 ml aliquot of each was pulled and frozen at -70°C to function as T0 material. The remaining material was held in a 25°C incubator. Consecutive 10 ml aliquots of each construct were pulled at 1, 2, 4, 8, and 12 weeks. Each aliquot was stored at -70°C to be used for further analytics.

### Particle Mixtures

AdC68 #1 development drug substance lot was separated by CsCl into low and high-density particle fractions. AEX was used to determine the viral particle concentration for each of the isolated bands. The concentration of the high-density band was 8.0E11 VP/mL and the concentration of the low-density band was 3.8E11 VP/mL. Both samples were diluted to 2E11 VP/mL to then make a range of empty and full particle mixtures at the same particle concentration. The samples were aliquoted at 1.5 ml volumes and frozen at –70°C to be used for analytical testing.

### AEX-HPLC of Stability Samples for A260/280 & Mixtures FLD + A260

The AEX-HPLC method is used to determine product purity and virus particle concentration. Relative retention time is also measured which correlates to changes in surface charge. In this case, a high affinity, anion exchange column (GE Healthcare, Resource Q, 1 ml 15 μm, 6.4 × 30 mm, Part No. 17-1177-01) was used to separate the AdC68 and impurities. AdC68 samples are diluted with formulation buffer and analyzed against a standard curve that runs from 0.25E11 VP/mL to 3.0E11 VP/mL. Elution of the bound molecules is achieved using a salt gradient with a flow rate of 1.0 ml/min over the course of 30 min. Molecules are analyzed using fluorescence detection with a 280 nm excitation and 320 nm emission, as well as UV detection at 260 nm. Relative purity is determined by comparing area counts of the eluting peaks and is reported as a percent of the total area for all peaks. Particle quantitation is achieved by correlation of the unknowns to a standard curve. Relative retention time (acidic shift) is measured by comparing the average elution time of all standards to that of the sample.

### Denaturing A260 & A280 Measurements

Adenovirus samples were disrupted via a 1% SDS solution for approximately 15 min. After denaturation, the samples are transferred to a cuvette and analyzed at 260 and 280 nm. The appropriate Beer’s Law equation is used to calculate concentration using the 260 nm measurement. A purity ratio is also calculated using both the 260 and 280 nm absorbances.

## Results

After construction, AdC68 #1 and #2 contained genomes of 34.8 and 36.4kb, respectively. Even though the manufacturing processes for these constructs were comparable, unique features appeared between them. Firstly, the AdC68 #1 per particle infectivity was about twice that of AdC68 #2 (data not shown). Secondly, manufacturing yields for AdC68 #2 campaigns were about twice that of AdC68 #1 (data not shown). Lastly, the constructs yielded different absorbances at 260 nm with similar particle numbers (data not shown) suggesting that genome packaging varied between them. Further investigation was initiated to ascertain viral particle attributes including integrity and content.

### Particle Content Investigation

Dynamic light scattering (DLS) and electron microscopy (EM) were employed to confirm particle integrity and both showed that the constructs contained uniform, monomeric viral particles of the expected diameter (data not shown).

**FIGURE 1:**
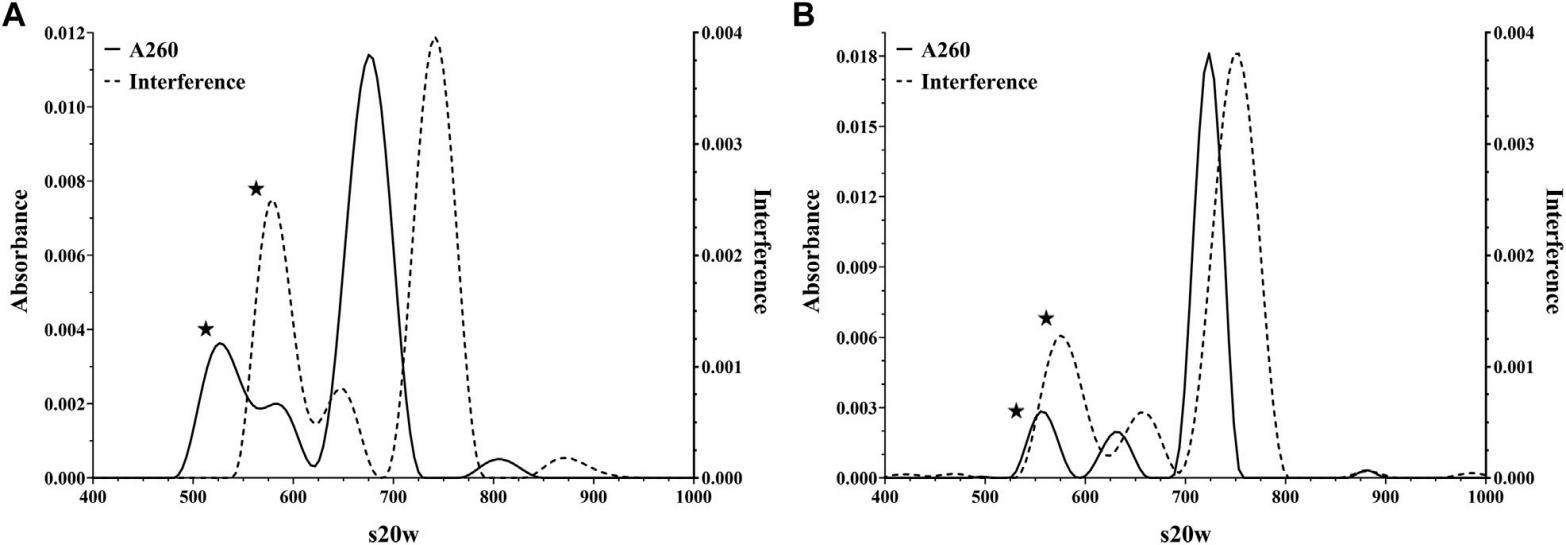
Representative Analytical Ultracentrifugation analyses of AdC68 construct #1 (Panel A) and #2 (Panel B) showing detection at both the absorbance (260 nm) and interference channels.

AUC, as the gold standard, and then DCS, as an orthogonal approach, were performed to examine the particle content. As shown in Figure 1, the AUC demonstrates that these materials contain capsids of various sedimentation coefficients consistent with a heterogeneous genomic content, and the constructs varied from each other in the fractional amount of each form (Table 1). In addition to leveraging the interference signal for quantitation, absorbance data at 260 nm were collected to give additional insight into capsid composition (i.e., capsids containing DNA would exhibit a stronger signal at 260 nm). As highlighted in Figure 1, the population with the smallest sedimentation coefficient exhibited a wide disparity between its absorbance and interference signals while the other three populations were in harmony. The fact that this population displayed lower absorbance relative to interference signal suggested these capsids likely did not contain a genome. These data, in addition to previous studies (Berkowitz and Philo, 2007; Berkowitz, 2008), supported the initial labeling of the capsid populations as empty, intermediate, full, and aggregates (Table 1).

**TABLE 1:** Analytical Ultracentrifugation and Differential Centrifugation Sedimentation quantification of AdC68 viral particle isoforms using interference and light scattering detection, respectively.

AdC68	AUC				DCS			
	Empty (%)	Intermediate (%)	Full (%)	Aggregates (%)	Empty (%)	Intermediate (%)	Full (%)	Aggregates (%)
#1	32	10	56	3	30	14	56	nd
#2	21	9	68	1	21	8	71	nd

“nd” denotes not detected.

As with the AUC interference channel, the light scattering employed by DCS (Bondoc and Fitzpatrick, 1998; Shih et al., 2010) is unbiased by the presence or absence of a genome. Therefore, when assessing particles of uniform size by DCS, the amount of light scattering is a direct correlation to particle number (Hohl et al., 2017). A qualitative comparison indicated a good alignment between DCS data and AUC interference data (Table 1). Furthermore, the DCS data also confirmed that AdC68 #1 contained a higher percentage of empty capsids than AdC68 #2 (Table 1).

Together, these data demonstrated that both constructs formed particles of appropriate size and shape, but AdC68 #1 contained significantly more low-density (empty) capsids than AdC68 #2. This trait was consistent among several batches of both constructs (data not shown) suggesting this was a feature of these embodiments.

### Empty/Full Capsid Isolation and Analysis

**FIGURE 2:**
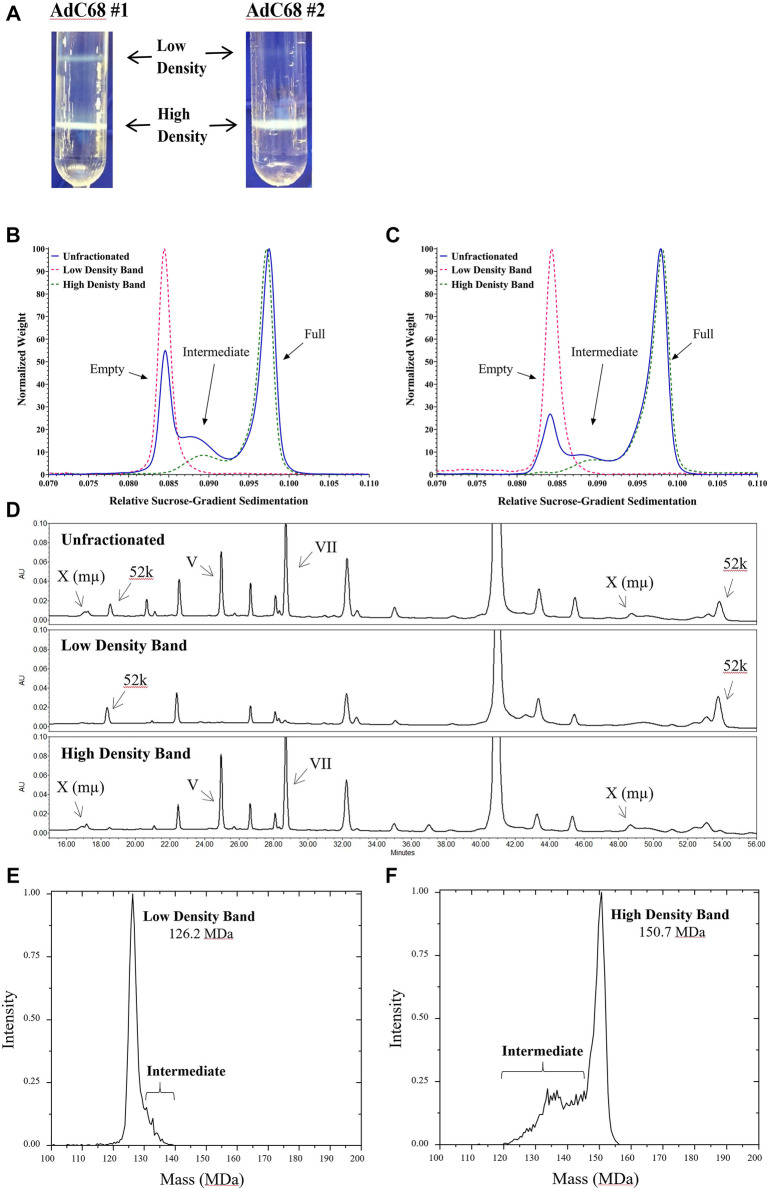
Analysis of preparative ultracentrifugation bands for AdC68 #1 and #2. Panel A is a representative picture of the CsCl preparative fractionation. Panels B and C are DCS overlays of the unfractionated and low/high density fractions for AdC68 #1 and #2, respectively. Panel D is a Reversed Phased High Performance Liquid Chromatography stacked-plot of the proteome content for AdC68 #1 unfractionated and low density/high density fractions. Panel E and Panel F are Charge Detection Mass Spectrometry spectra of the AdC68 #1 low/high density fractions showing the experimentally determined molecular masses, respectively.

Since column chromatography was used as the primary purification technique for these materials, as opposed to density centrifugation, the presence of genome packaging variants in the final material was unsurprising. To further study these variants and confirm the initial peak identifications, materials were fractionated by CsCl density ultracentrifugation (Prage et al., 1972; Burlingham et al., 1974; Daniell, 1976; Tibbetts and Giam, 1979; Tóth et al., 1982; Vellekamp et al., 2001; Sutjipto et al., 2005). The net result was two viral particle populations aptly labeled as the low-density band and the high-density band (Figure 2A). Presumably these represented primarily “genome-free or empty” capsids and the “genome-containing or full/mature” capsids, respectively.

The fractions were extracted from the CsCl gradient and evaluated. A negative staining EM investigation showed the typical patterns (Sutjipto et al., 2005) seen for empty and full/mature capsids, respectively (data not shown). Tests for infectivity (Table 2) showed that the particles from the low-density fraction exhibited extremely low infectivity as expected (Burlingham et al., 1974; Daniell, 1976) while the particles from the high-density fraction displayed infectivity ratios in line with expectations for the product and recombinant chimpanzee adenovirus (Roy et al., 2004). DCS analysis (Figure 2B, C) revealed that the particles from the low-density fraction aligned with the previously labeled empty capsid region and that the particles from the high-density fraction contained both the previously labeled intermediate and full capsid region.

**TABLE 2:** Infectivity of both the low/high density viral particle fractions for AdC68 #1 and #2.

AdC68	IFU/mL	Vp/IFU
#1	Low Density Particles	251,966^a^
	High Density Particles	55
#2	Low Density Particles	19,234^a^
	High Density Particles	123

a Extrapolated beyond calibrated method range.

Reversed phase high performance liquid chromatography (RP-HPLC) (Vellekamp et al., 2001; Lehmberg et al., 1999; Chelius et al., 2002; Takahashi et al., 2006) of AdC68 #1 (Figure 2D) revealed that the low-density material contained the 52 k protein which is known to be associated only with empty capsids (Sutjipto et al., 2005; Condezo et al., 2015; Ahi and Mittal, 2016) while the high-density material contained proteins V, VII and X, all of which are hallmarks for mature/full capsids (Ahi and Mittal, 2016). Protein identifications were consistent with internal LC-MS characterization and profiles observed in literature (Lehmberg et al., 1999; Chelius et al., 2002; Takahashi et al., 2006). AdC68 #2 had similar results (data not shown).

Finally, CDMS was employed to determine the masses of the low and high-density fractions and confirm the presence of empty and fully packaged capsids. Figure 2E, F show the CDMS spectra for the low-density and high-density bands of Ad68 #1, respectively. The primary mass of the high-density band is 150.7 MDa, which is in range with the generally accepted mass for adenovirus mature/full particles of 150 MDa (van Oostrum and Burnett, 1985; Rux and Burnett, 2004). The theoretical mass of an adenoviral particle without a genome is ∼127 MDa which is in close alignment to the primary mass of the low-density band: 126.2 MDa. As with DCS, the CDMS data suggests that the high-density fraction contains a majority of the intermediate capsid species. Furthermore, these data support the assignments of empty, intermediate, and full capsids in Figure 2C and Figure 2D.

Together these data definitively establish that the high-density and low-density bands primarily contain capsids with and without genomes, respectively. However, neither population was 100% homogeneous. The preparation of these highly enriched materials offered the opportunity to investigate the capabilities of different analytical tools to quantify viral particles with varied numbers of empty and full capsids.

### Empty/Full Capsid Mixture and Viral Particle Quantification Study

To estimate the impact of empty particles on particle content methods, a series of mixtures of the low and high-density materials were produced and evaluated by several analytical methods. AEX-HPLC was used to measure the viral particle titers for AdC68 #1 100% low-density and 100% high-density parent fractions. Based on those concentrations, theoretical mixtures were created as listed in Table 3. Those mixtures were then analyzed on DCS to determine relative percentages of empty, intermediate, and mature/full species. As shown in Table 3, the output from the DCS confirmed the targeted mixtures. Since DCS signal intensity is a direct readout of total particle number, the viral particle concentrations of the parent low and high-density fractions measured by AEX-HPLC using intrinsic protein fluorescence must have been accurately determined.

**TABLE 3:** Differential Centrifugation Sedimentation analyses and 260/280 absorbance measurements of AdC68 #1 low/high density viral particle fractions as well as mixtures of said fractions.

Mixture	DCS band percentage			Absorbance units		
	Empty	Intermediate	Full	260 nm	280 nm	Ratio
100% High Density	nd	10.8	89.2	0.234	0.179	1.307
90% High & 10% Low Density	8.9	10.9	80.2	0.209	0.161	1.299
80% High & 20% Low Density	19.4	9.7	71.0	0.199	0.158	1.265
70% High & 30% Low Density	31.6	7.6	60.8	0.175	0.145	1.204
60% High & 40% Low Density	40.0	7.6	52.4	0.162	0.141	1.154
50% High & 50% Low Density	51.0	5.4	43.6	0.152	0.137	1.112
40% High & 60% Low Density	60.3	5.0	34.7	0.140	0.133	1.047
30% High & 70% Low Density	69.2	3.0	25.9	0.123	0.124	0.990
20% High & 80% Low Density	79.5	3.6	16.9	0.106	0.117	0.908
10% High & 90% Low Density	88.4	3.3	8.3	0.092	0.111	0.830
100% Low Density	100	nd	nd	0.080	0.108	0.746

“nd” denotes not detected.

To finalize the data set, denaturing A260/280 nm measurements were performed as described (Materials and Methods). As shown in Table 3, A260/280 ratio increases from 0.746 up to 1.307. These values were consistent with adenoviral preparations containing primarily empty/immature to mature/full viral capsids (Prage et al., 1972; Burlingham et al., 1974; Daniell, 1976; Tóth et al., 1982; Vellekamp et al., 2001; Sweeney and Hennessey, 2002; Sutjipto et al., 2005; Berkowitz, 2008). Interestingly, the raw denaturing A260 value increases almost 3-fold over the range of samples which are known to have the same viral particle number (Table 3).

**FIGURE 3:**
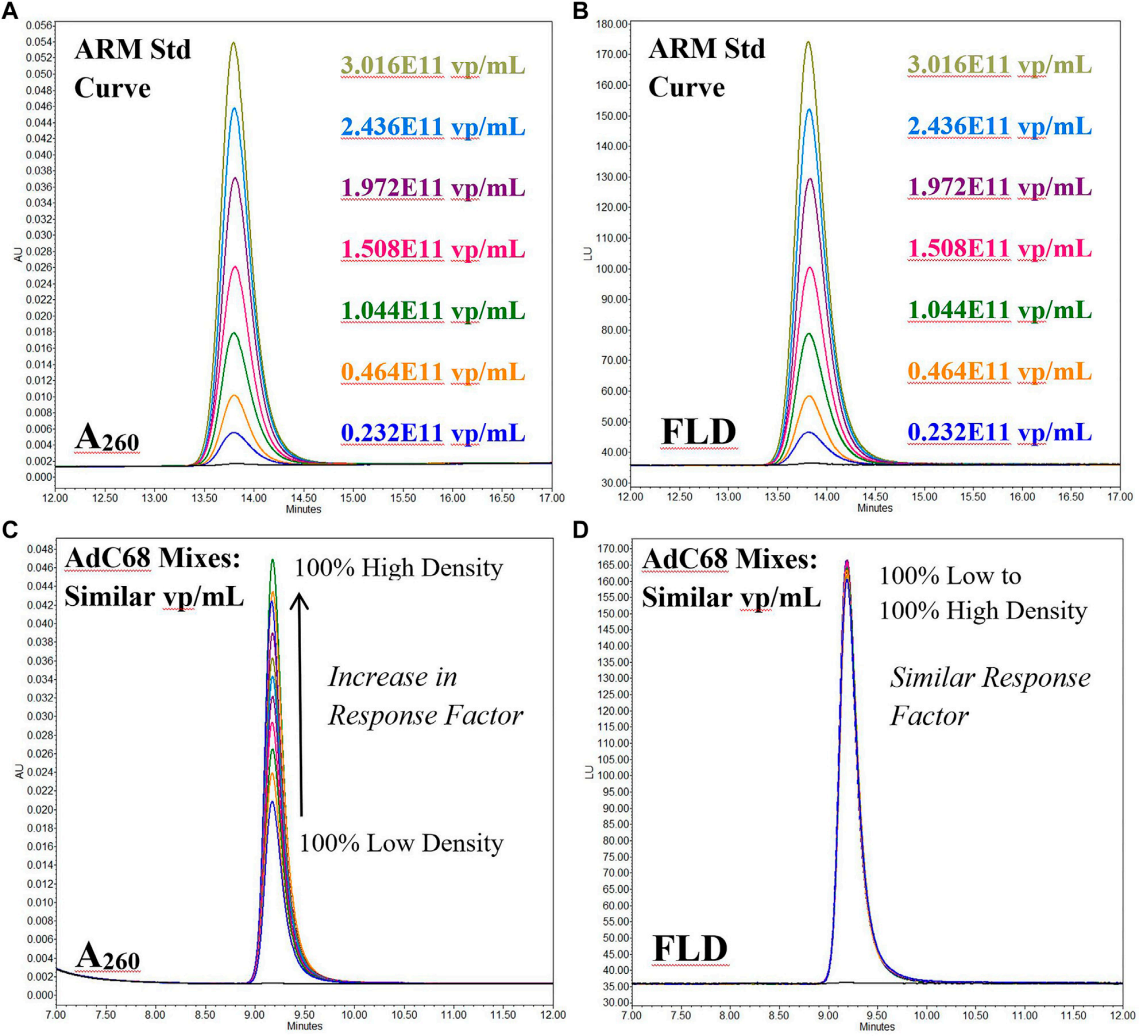
Anion Exchange High Performance Liquid Chromatography overlays of the fluorescence (FLD) and 260 nm absorbance (A_260_) channels for both Human Ad5 Reference Material (Panels A & B) and mixtures of AdC68 low/high density viral particle fractions (Panels C & D). The Human Ad5 Reference Material data is a serial dilution and shows a similar response factor for both channels. The AdC68 mixtures give an increasing response factor on the 260 nm channel for samples of the similar particle # but differing particle density while that for the fluorescence channel is consistent among all samples.

This bias can also be observed in overlays of the A260 and fluorescence channels of AEX-HPLC intact particle analyses (Figure 3). As expected for the human ARM which is known to contain a high percentage of packaged capsids (Hutchins et al., 2000; Hutchins, 2002; Berkowitz, 2008), both the A260 (Figure 3A) and fluorescence AEX-HPLC (Figure 3B) channels show commensurate, concentration-dependent increase in signal. However, when the empty/full capsid mixtures (known to contain similar viral particle titers) were studied, the response factors for these two detection modes did not align (Figure 3C, D). For all mixtures, signal from the fluorescence channel was concordant while that from the A260 channel increased in proportion to the percentage of high-density particles. The overall ∼2.5 fold increase in response was comparable to that of the denaturing A260 measurement with the difference between them probably related to light scattering effects from analyses of the intact particles.

Based on this data, application of the denaturing 260 nm based concentration calculation is not sufficient for determining the particle titer of the adenovirus constructs used in this study, primarily due to the large abundance of empty capsids.

### Viral Particle Stability Study

**FIGURE 4:**
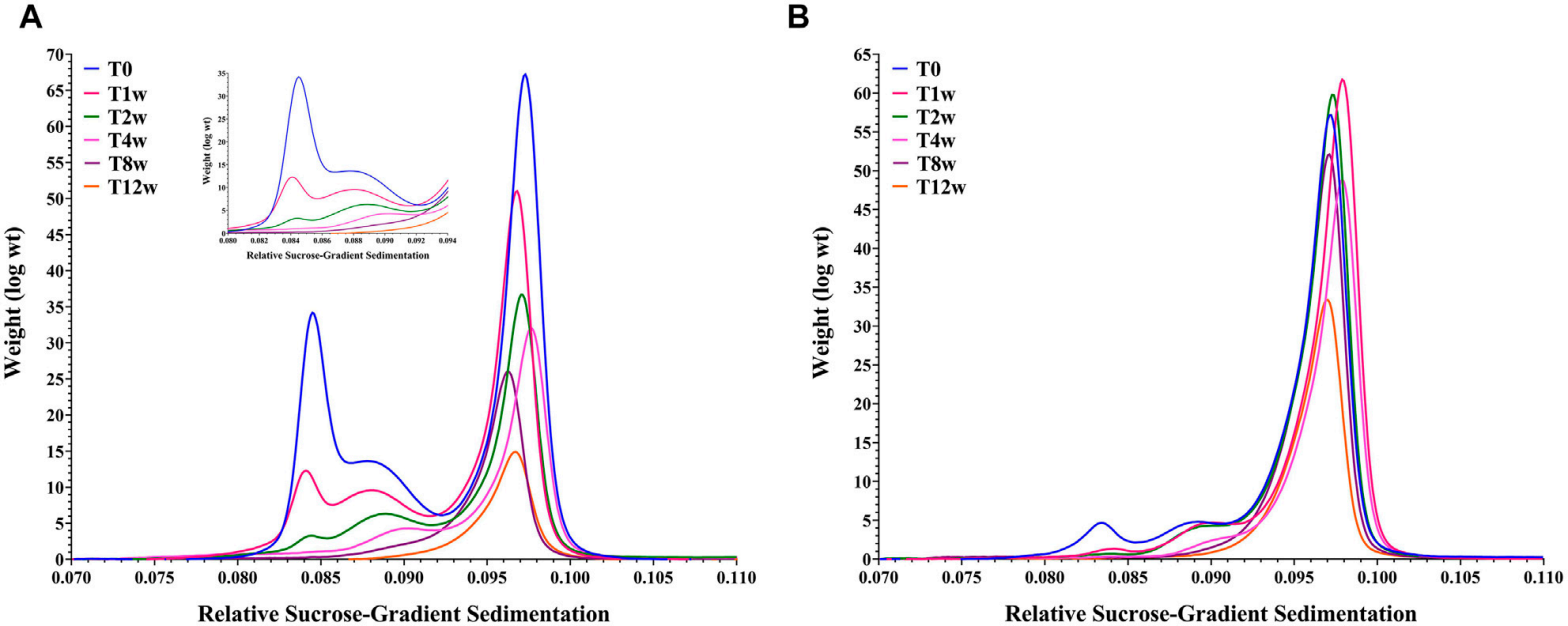
Overlay of Differential Centrifugation Sedimentation data for an AdC68 #1 (Panel A) and AdC68 #2 (Panel B) accelerated stability time course.

In addition to assessing particle content, an additional aim was to determine how genome content and size impacted capsid stability. The relative stability for these two materials was explored through an accelerated stability hold followed by DCS and AEX-HPLC time-point measurements. Figure 4A, B clearly show that the empty capsid population decreased dramatically such that by the end of the second week of the study, only a fractional amount of that species remained. Limited stability of empty capsids compared to full capsids is consistent with previous observations (Prage et al., 1972; Burlingham et al., 1974; Tibbetts and Giam, 1979; Tóth et al., 1982).

The stability profile of the fully packaged capsid populations deviate significantly. AdC68 #1 exhibited a steady decrease in intact particle number and infectivity (data not shown) such that only ∼25% of the initial capsid number remained after 12 weeks (Figure 4A). In contrast, AdC68 #2 was more resistant to degradation, taking up to 4 weeks before any discernable particle loss was detected. By 12 weeks, > 50% of the particles remain intact (Figure 4B) and infectious (data not shown).

The AEX-HPLC measurements showed a biphasic result for viral particle titer consistent with rapid depletion of empty capsids followed by a gradual disappearance of the full capsids (data not shown). These data also indicated that breakdown of AdC68 #2 particles was at a reduced rate compared to AdC68 #1 (data not shown).

Both data sets were consistent and indicated these AdC68 builds show differential stability profiles. Interestingly, these data convey another deficiency in the denaturing A260 concentration method compared to AEX-HPLC. Because the viral particles are disrupted prior to quantification, the A260 method is blind to degradation and not a useful measurement of stability.

## Discussion

Construction of a recombinant, replication deficient adenovirus is a delicate operation. A balance must be struck between removal of the replication machinery and insertion of the therapeutic material while maintaining a genome size optimal for packaging and stability (Saha et al., 2014). These two constructs exhibited differences in infectivity ratios, stability profiles, production yields and genome packaging: an outcome which is almost certainly due, at least in part, to divergent genome lengths. However, in many ways, the variability in quality attributes between constructs is inconsequential insomuch as target profiles for the individual builds can be maintained. To maintain said profile, an analytical package must be developed that can measure the associated quality attributes. This study focused on two attributes specifically, capsid content and viral particle concentration.

### Viral Particle Content Analysis (Ie. Genome Packaging Profile)

The majority of the viral particle content tools presented in this manuscript harken back to the past 50 or so years of adenovirus characterization. From the early days of density gradient ultracentrifugation to separate differentially packaged capsids followed by SDS-PAGE to examine the protein content (Prage et al., 1972; Burlingham et al., 1974; Daniell, 1976; Tibbetts and Giam, 1979; Tóth et al., 1982), to substitution of SDS-PAGE with RP-HPLC and mass spectrometry (Lehmberg et al., 1999; Vellekamp et al., 2001; Chelius et al., 2002; Takahashi et al., 2006; Benevento et al., 2014; Perez-Berna et al., 2014; van Tricht et al., 2018) and rounding out with direct content analysis via AUC and DCS (Bondoc and Fitzpatrick, 1998; Berkowitz and Philo, 2007; Berkowitz, 2008; Yang et al., 2008; Shih et al., 2010). The outlier is the introduction of direct measurement of adenoviral mass via CDMS, something that would have been thought almost impossible just a few years ago.

When it comes to direct viral particle content analysis, AUC is the current gold standard, however, it has drawbacks including low sample throughput, high volume sample burn, and complex mathematical data analyses. In this study, DCS gave comparable results while being a more user-friendly technique for daily capsid content analysis. DCS gives results in ∼15 min using ∼100 µl of material.

CDMS, though currently conducted on prototype instrumentation, is gaining in popularity as a biotherapeutic characterization tool (Pansieri et al., 2020; Wörner et al., 2020; Miller et al., 2021). As with DCS, CDMS is able to obtain information using relatively low volumes of material, an advantage for viral applications. While CDMS was not used to quantitate particle content for the mixtures in this study, the data included represent a proof of concept that the technology could be used for such purposes. A similar study was performed on AAV capsids, which demonstrated that CDMS was highly complementary to AUC (Werle et al., 2021).

### Viral Particle Concentration Determination

Even though the exact number of critical quality attributes associated with any pharmaceutical product may vary, all must include a measure of quantity (Center for Drug Evaluatio, 1999), often called strength or concentration, that can be determined accurately throughout product lifetime. The historical approach for adenovirus utilizes UV absorbance at 260 nm of denatured particles via the extinction coefficient determined by Maizel et al. (1968b). Though the exact value of the extinction coefficient has been revisited (Sweeney and Hennessey, 2002), the ease of this determination has made it a staple in adenoviral laboratories.

All told, while the denaturing 260 nm based concentration calculation is sufficient for initial viral particle titering for materials like human ARM (Berkowitz, 2008) and other constructs with a low percentage of empty capsids, the accuracy of this approach wanes as the percentage of empty capsids increases. Thus, in the pharmaceutical space, adequate understanding of the properties of the adenovirus material should be understood before attempting to use the A260 measurement. Specifically, adenovirus applications that have empty and intermediate capsids should avoid use of the A260 measurement or at minimum complement that analyses with AEX-HPLC quantification.

To avoid the empty capsid interference, dose and titering can be determined by counting viral genomes using PCR tools (Thomas et al., 2007; Crettaz et al., 2008; Gallaher and Berk, 2013). Though these methods are more specific in terms of determining DNA amount, they can be biased due to off-targeting of truncated genomes (intermediate particles) that may contain the chosen PCR amplicon. In addition, as with denaturing A260, information about particle integrity is lost so these measurements have little value monitoring particle stability.

In general, a technique that is not biased from the particle content should be implemented for viral particle titer measurements. This allows for consistent particle quantification, regardless of the construct design or implementation of density or charge based purification. The AEX-HPLC method described in this manuscript is a robust tool for accurately counting capsids because the fluorescence detection is blind to viral particle maturity. The one drawback to this method is that it is necessary to have reference standard to quantify against. To this end, the work of the Adenovirus Reference Material Working Group (Hutchins et al., 2000; Hutchins, 2002) created a singular standard to which all other materials can be bridged.

## Conclusion

From the early days of its study, adenovirus was shown to generate heterogenous packaging populations (Smith, 1965a; Smith, 1965b; Maizel et al., 1968a). This observation generated numerous studies of adenoviral particle maturation leading to a well defined model for capsid assembly (Ahi and Mittal, 2016). However, unlike adeno-associated virus-based gene therapies, where capsid genome packaging ratio is of particular interest because of its clinical significance (Wright, 2008; Wright, 2014a; Wright, 2014b), its impact on adenovirus has had less scrutiny. Here we show that at minimum, this ratio can have considerable influence on viral particle titering depending on the method chosen. It will also impact the stability and infectivity profiles. In total, a comprehensive adenoviral analytical control strategy is not complete without a robust accounting of the capsid packaging profile.

## Data Availability

The datasets presented in this article are not readily available because Raw data may be deemed proprietary by legal. Requests to access the datasets should be directed to lawrence.thompson@pfizer.com.

## Abbreviations

AUC, analytical ultracentrifugation; DCS, differential centrifugation sedimentation; CDMS, charge detection mass spectrometry; ARM, adenovirus reference material; A260, absorbance at 260 nm; UV, ultra-violet; AEX-HPLC, anion-exchange high performance liquid chromatography; DNA, deoxyribonucleic acids; qPCR, quantitative polymerase chain reaction; DLS, dynamic light scattering; EM, electron microscopy; RPM, revolutions per minute; CsCl, cesium chloride; mL, milliliters; HEK293, cell line derived from human embryonic kidney cells; IFU, infectivity units; VP, viral particles; RP-HPLC, reversed-phase high performance liquid chromatography; ACN, acetonitrile; TFA, trifluoroacetic acid; ELIT, electrostatic linear ion trap; FFT, fast Fourier transforms; MDa, megadalton; mM, millimolar; SDS, sodium dodecyl sulfate; kb, kilobases; LC-MS, liquid chromatography coupled to mass spectrometry.

## References

[B1] Ahi Y. S. Mittal S. K. (2016). Components of Adenovirus Genome Packaging. Front. Microbiol. 7, 1503. 10.3389/fmicb.2016.01503 27721809 PMC5033970

[B2] Benevento M. Di Palma S. Snijder J. Moyer C. L. Reddy V. S. Nemerow G. R. (2014). Adenovirus Composition, Proteolysis, and Disassembly Studied by In-Depth Qualitative and Quantitative Proteomics. J. Biol. Chem. 289 (16), 11421–11430. 10.1074/jbc.m113.537498 24591515 PMC4036278

[B3] Berkowitz S. A. (2008). Determining the Concentration and the Absorptivity Factor at 260 Nm in Sodium Dodecyl Sulfate of the Adenovirus Reference Material Using Analytical Ultracentrifugation. Anal. Biochem. 380 (1), 152–154. 10.1016/j.ab.2008.05.014 18539129

[B4] Berkowitz S. A. Philo J. S. (2007). Monitoring the Homogeneity of Adenovirus Preparations (A Gene Therapy Delivery System) Using Analytical Ultracentrifugation. Anal. Biochem. 362 (1), 16–37. 10.1016/j.ab.2006.11.031 17223062

[B5] Blanche F. Cameron B. Barbot A. Ferrero L. Guillemin T. Guyot S. (2000). An Improved Anion-Exchange HPLC Method for the Detection and Purification of Adenoviral Particles. Gene Ther. 7 (12), 1055–1062. 10.1038/sj.gt.3301190 10871755

[B6] Bondoc L. Fitzpatrick S. (1998). Size Distribution Analysis of Recombinant Adenovirus Using Disc Centrifugation. JIMB 20, 317–322. 10.1038/sj.jim.2900529

[B7] Burlingham B. T. Brown D. T. Doerfler W. (1974). Incomplete Particles of Adenovirus. I. Characteristics of the DNA Associated with Incomplete Adenovirions of Types 2 and 12. Virology 60 (2), 419–430. 10.1016/0042-6822(74)90336-5 4844422

[B8] Capone S. D’Alise A. M. Ammendola V. Colloca S. Cortese R. Nicosia A. (2013). Development of Chimpanzee Adenoviruses as Vaccine Vectors: Challenges and Successes Emerging from Clinical Trials. Expert Rev. Vaccin. 12 (4), 379–393. 10.1586/erv.13.15 23560919

[B9] Center for Drug Evaluation and Research, ICH, Q6B Specifications: Test Procedures and Acceptance Criteria for Biotechnological/Biological Products. 1999, International Conference on Harmonisation of Technical Requirements for Registration of Pharmaceuticals for Human Use.

[B10] Chelius D. Hühmer A. F. R. Shieh C. H. Lehmberg E. Traina J. A. Slattery T. K. (2002). Analysis of the Adenovirus Type 5 Proteome by Liquid Chromatography and Tandem Mass Spectrometry Methods. J. Proteome Res. 1 (6), 501–513. 10.1021/pr025528c 12645618

[B11] Cheng C. Wang L. Ko S.-Y. Kong W.-P. Schmidt S. D. Gall J. G. D. (2015). Combination Recombinant Simian or Chimpanzee Adenoviral Vectors for Vaccine Development. Vaccine 33 (51), 7344–7351. 10.1016/j.vaccine.2015.10.023 26514419 PMC11059210

[B12] Cohen C. J. Xiang Z. Q. Gao G. P. Ertl H. C. J. Wilson J. M. Bergelson J. M. , Chimpanzee Adenovirus CV-68 Adapted as a Gene Delivery Vector Interacts with the Coxsackievirus and Adenovirus Receptor *.* J. Gen. Virol., 2002. 83(Pt 1): p. 151–155. 10.1099/0022-1317-83-1-151 11752711

[B13] Condezo G. N. Marabini R. Ayora S. Carazo J. M. Alba R. Chillón M. (2015). Structures of Adenovirus Incomplete Particles Clarify Capsid Architecture and Show Maturation Changes of Packaging Protein L1 52/55k. J. Virol. 89 (18), 9653–9664. 10.1128/jvi.01453-15 26178997 PMC4542391

[B14] Contino N. C. Jarrold M. F. (2013). Charge Detection Mass Spectrometry for Single Ions with a Limit of Detection of 30 Charges. Int. J. Mass Spectrom. 345-347, 153–159. 10.1016/j.ijms.2012.07.010

[B15] Crettaz J. Olague C. Vales A. Aurrekoetxea I. Berraondo P. Otano I. (2008). Characterization of High-Capacity Adenovirus Production by the Quantitative Real-Time Polymerase Chain Reaction: a Comparative Study of Different Titration Methods. J. Gene Med. 10 (10), 1092–1101. 10.1002/jgm.1236 18642400

[B16] Daniell E. (1976). Genome Structure of Incomplete Particles of Adenovirus. J. Virol. 19 (2), 685–708. 10.1128/jvi.19.2.685-708.1976 957486 PMC354903

[B17] Draper B. E. Anthony S. N. Jarrold M. F. (2018). The FUNPET-A New Hybrid Ion Funnel-Ion Carpet Atmospheric Pressure Interface for the Simultaneous Transmission of a Broad Mass Range. J. Am. Soc. Mass. Spectrom. 29 (11), 2160–2172. 10.1007/s13361-018-2038-3 30112619

[B18] Draper B. E. Jarrold M. F. (2019). Real-Time Analysis and Signal Optimization for Charge Detection Mass Spectrometry. J. Am. Soc. Mass. Spectrom. 30 (6), 898–904. 10.1007/s13361-019-02172-z 30993638

[B19] Evans R. K. Nawrocki D. K. Isopi L. A. Williams D. M. Casimiro D. R. Chin S. (2004). Development of Stable Liquid Formulations for Adenovirus-Based Vaccines. J. Pharm. Sci. 93 (10), 2458–2475. 10.1002/jps.20157 15349956

[B20] Ewer K. J. Barrett J. R. Belij-Rammerstorfer S. Sharpe H. Makinson R. Morter R. (2021). T Cell and Antibody Responses Induced by a Single Dose of ChAdOx1 nCoV-19 (AZD1222) Vaccine in a Phase 1/2 Clinical Trial. Nat. Med. 27 (2), 270–278. 10.1038/s41591-020-01194-5 33335323

[B21] Farina S. F. Gao G.-p. Xiang Z. Q. Rux J. J. Burnett R. M. Alvira M. R. (2001). Replication-defective Vector Based on a Chimpanzee Adenovirus. J. Virol. 75 (23), 11603–11613. 10.1128/jvi.75.23.11603-11613.2001 11689642 PMC114747

[B22] Folegatti P. M. Ewer K. J. Aley P. K. Angus B. Becker S. Belij-Rammerstorfer S. (2020). Safety and Immunogenicity of the ChAdOx1 nCoV-19 Vaccine against SARS-CoV-2: a Preliminary Report of a Phase 1/2, Single-Blind, Randomised Controlled Trial. Lancet 396 (10249), 467–478. 10.1016/S0140-6736(20)31604-4 32702298 PMC7445431

[B23] Gallaher S. D. Berk A. J. (2013). A Rapid Q-PCR Titration Protocol for Adenovirus and Helper-dependent Adenovirus Vectors that Produces Biologically Relevant Results. J. Virol. Methods 192 (1-2), 28–38. 10.1016/j.jviromet.2013.04.013 23624118 PMC3679352

[B24] Hohl A. Ramms A. S. Dohmen C. Mantwill K. Bielmeier A. Kolk A. (2017). Adenovirus Particle Quantification in Cell Lysates Using Light Scattering. Hum. Gene Ther. Methods 28 (5), 268–276. 10.1089/hgtb.2017.052 28806885

[B25] Hutchins B. (2002). Development of a Reference Material for Characterizing Adenovirus Vectors. Bioprocess J. 1 (1), 25–29. 10.12665/j11.hutchins

[B26] Hutchins B. Sajjadi N. Seaver S. Shepherd A. Bauer S. R. Simek S. (2000). Working toward an Adenoviral Vector Testing Standard. Mol. Ther. 2 (6), 532–534. 10.1006/mthe.2000.0217 11124052

[B27] Klyushnichenko V. Bernier A. Kamen A. Harmsen E. (2001). Improved High-Performance Liquid Chromatographic Method in the Analysis of Adenovirus Particles. J. Chromatogr. B Biomed. Sci. Appl. 755 (1-2), 27–36. 10.1016/s0378-4347(00)00597-1 11393714

[B28] Kondylis P. Schlicksup C. J. Zlotnick A. Jacobson S. C. (2019). Analytical Techniques to Characterize the Structure, Properties, and Assembly of Virus Capsids. Anal. Chem. 91 (1), 622–636. 10.1021/acs.analchem.8b04824 30383361 PMC6472978

[B29] Kuhn I. Larsen B. Gross C. Hermiston T. (2007). High-performance Liquid Chromatography Method for Rapid Assessment of Viral Particle Number in Crude Adenoviral Lysates of Mixed Serotype. Gene Ther. 14 (2), 180–184. 10.1038/sj.gt.3302851 17024108

[B30] Ledgerwood J. E. DeZure A. D. Stanley D. A. Coates E. E. Novik L. Enama M. E. (2017). Chimpanzee Adenovirus Vector Ebola Vaccine. N. Engl. J. Med. 376 (10), 928–938. 10.1056/nejmoa1410863 25426834

[B31] Lehmberg E. Traina J. A. Chakel J. A. Chang R.-J. Parkman M. McCaman M. T. (1999). Reversed-phase High-Performance Liquid Chromatographic Assay for the Adenovirus Type 5 Proteome. J. Chromatogr. B: Biomed. Sci. Appl. 732 (2), 411–423. 10.1016/s0378-4347(99)00316-3 10517364

[B32] Maizel J. V. Jr. White D. O. Scharff M. D. (1968a). The Polypeptides of Adenovirus II. Soluble proteins, cores, top components and the structure of the virion. Virology 36 (1), 126–136. 10.1016/0042-6822(68)90122-0 5669983

[B33] Maizel J. V. Jr. White D. O. Scharff M. D. (1968b). The Polypeptides of Adenovirus. I. Evidence for Multiple Protein Components in the Virion and a Comparison of Types 2, 7A, and 12. Virology 36 (1), 115–125. 10.1016/0042-6822(68)90121-9 5669982

[B34] Mangel W. San Martín C. (2014). Structure, Function and Dynamics in Adenovirus Maturation. Viruses 6 (11), 4536–4570. 10.3390/v6114536 25421887 PMC4246237

[B35] Miller L. M. Barnes L. F. Raab S. A. Draper B. E. El-Baba T. J. Lutomski C. A. (2021). Heterogeneity of Glycan Processing on Trimeric SARS-CoV-2 Spike Protein Revealed by Charge Detection Mass Spectrometry. J. Am. Chem. Soc. 143 (10), 3959–3966. 10.1021/jacs.1c00353 33657316 PMC8543487

[B36] Pansieri J. Iashchishyn I. A. Fakhouri H. Ostojić L. Malisauskas M. Musteikyte G. (2020). Templating S100A9 Amyloids on Aβ Fibrillar Surfaces Revealed by Charge Detection Mass Spectrometry, Microscopy, Kinetic and Microfluidic Analyses. Chem. Sci. 11 (27), 7031–7039. 10.1039/c9sc05905a 34122996 PMC8159403

[B37] Perez-Berna A. J. Mangel W. F. McGrath W. J. Graziano V. Flint J. San Martin C. (2014). Processing of the L1 52/55k Protein by the Adenovirus Protease: a New Substrate and New Insights into Virion Maturation. J. Virol. 88 (3), 1513–1524. 10.1128/jvi.02884-13 24227847 PMC3911601

[B38] Pierson E. E. Keifer D. Z. Asokan A. Jarrold M. F. (2016). Resolving Adeno-Associated Viral Particle Diversity with Charge Detection Mass Spectrometry. Anal. Chem. 88 (13), 6718–6725. 10.1021/acs.analchem.6b00883 27310298 PMC6537880

[B39] Prage L. Höglund S. Philipson L. (1972). Structural Proteins of Adenoviruses 8. Characterization of incomplete particles of adenovirus type 3. Virology 49 (3), 745–757. 10.1016/0042-6822(72)90531-4 5072634

[B40] Putter J. S. (2021). Immunotherapy for COVID-19: Evolving Treatment of Viral Infection and Associated Adverse Immunological Reactions. Transfus. Apher. Sci. 60 (2), 103093. 10.1016/j.transci.2021.103093 33610448 PMC7881713

[B41] Roy S. Gao G. Lu Y. Zhou X. Lock M. Calcedo R. (2004). Characterization of a Family of Chimpanzee Adenoviruses and Development of Molecular Clones for Gene Transfer Vectors. Hum. Gene Ther. 15 (5), 519–530. 10.1089/10430340460745838 15144581

[B42] Rux J. J. Burnett R. M. (2004). Adenovirus Structure. Hum. Gene Ther. 15 (12), 1167–1176. 10.1089/hum.2004.15.1167 15684694

[B43] Saha B. Wong C. Parks R. (2014). The Adenovirus Genome Contributes to the Structural Stability of the Virion. Viruses 6 (9), 3563–3583. 10.3390/v6093563 25254384 PMC4189039

[B44] Shabram P. W. Giroux D. D. Goudreau A. M. Gregory R. J. Horn M. T. Huyghe B. G. (1997). Analytical Anion-Exchange HPLC of Recombinant Type-5 Adenoviral Particles. Hum. Gene Ther. 8 (4), 453–465. 10.1089/hum.1997.8.4-453 9054520

[B45] Shih S.-J. Yagami M. (2010). Validation of a Quantitative Method for Detection of Adenovirus Aggregation. Bioprocessing J. 9 (2), 25–33. 10.12665/j92.shih

[B46] Smith K. O. (1965). Studies on Adenovirus-12. I. Quantitative Correlations between Some Physical, Antigenic and Infectious Properties. J. Immunol. 94, 976–989. 14321442

[B47] Smith K. O. (1965). Cyclic Structure of Adenovirus DNA. Science 148 (3666), 100–102. 10.1126/science.148.3666.100 14258723

[B48] Stanley D. A. Honko A. N. Asiedu C. Trefry J. C. Lau-Kilby A. W. Johnson J. C. (2014). Chimpanzee Adenovirus Vaccine Generates Acute and Durable Protective Immunity against Ebolavirus challenge. Nat. Med. 20 (10), 1126–1129. 10.1038/nm.3702 25194571

[B49] Sutjipto S. Ravindran S. Cornell D. Liu Y.-H. Horn M. Schluep T. (2005). Characterization of Empty Capsids from a Conditionally Replicating Adenovirus for Gene Therapy. Hum. Gene Ther. 16 (1), 109–125. 10.1089/hum.2005.16.109 15703494

[B50] Sweeney J. A. Hennessey J. P. Jr. (2002). Evaluation of Accuracy and Precision of Adenovirus Absorptivity at 260 Nm under Conditions of Complete DNA Disruption. Virology 295 (2), 284–288. 10.1006/viro.2002.1406 12033787

[B51] Takahashi E. Cohen S. L. Tsai P. K. Sweeney J. A. (2006). Quantitation of Adenovirus Type 5 Empty Capsids. Anal. Biochem. 349 (2), 208–217. 10.1016/j.ab.2005.11.014 16360111

[B52] Tatsis N. Tesema L. Robinson E. R. Giles-Davis W. McCoy K. Gao G. P. (2006). Chimpanzee-origin Adenovirus Vectors as Vaccine Carriers. Gene Ther. 13 (5), 421–429. 10.1038/sj.gt.3302675 16319951

[B53] Thomas M. A. Lichtenstein D. L. Krajcsi P. Wold W. S. (2007). A Real-Time PCR Method to Rapidly Titer Adenovirus Stocks. Methods Mol. Med. 130, 185–192. 10.1385/1-59745-166-5:185 17401173

[B54] Tibbetts C. Giam C. Z. (1979). *In Vitro* association of Empty Adenovirus Capsids with Double-Stranded DNA. J. Virol. 32 (3), 995–1005. 10.1128/jvi.32.3.995-1005.1979 513209 PMC525949

[B55] Tóth M. Taródi B. Béládi I. (1982). Preparative Separation of Intact Incomplete and Empty Adenovirus Type 2 Particles. Acta Virol. 26 (4), 217–220. 6182779

[B56] van Oostrum J. Burnett R. M. (1985). Molecular Composition of the Adenovirus Type 2 Virion. J. Virol. 56 (2), 439–448. 10.1128/jvi.56.2.439-448.1985 4057357 PMC252598

[B57] van Tricht E. de Raadt P. Verwilligen A. Schenning M. Backus H. Germano M. (2018). Fast, Selective and Quantitative Protein Profiling of Adenovirus-Vector Based Vaccines by Ultra-performance Liquid Chromatography. J. Chromatogr. A 1581-1582, 25–32. 10.1016/j.chroma.2018.10.045 30389208 PMC7094600

[B58] Vellekamp G. Porter F. W. Sutjipto S. Cutler C. Bondoc L. Liu Y.-H. (2001). Empty Capsids in Column-Purified Recombinant Adenovirus Preparations. Hum. Gene Ther. 12 (15), 1923–1936. 10.1089/104303401753153974 11589834

[B59] Werle A. Powers T. W. Zobel J. F. Wappelhorst C. N. Jarrold M. F. Lyktey N. A. (2021). Comparison of Analytical Techniques to Quantitate the Capsid Content of Adeno-Associated Viral Vectors. Mol. Ther. Methods Clin. Dev. 23, 254–262. 10.1016/j.omtm.2021.08.009 34703846 PMC8505359

[B60] Whitfield R. J. Battom S. E. Barut M. Gilham D. E. Ball P. D. (2009). Rapid High-Performance Liquid Chromatographic Analysis of Adenovirus Type 5 Particles with a Prototype Anion-Exchange Analytical Monolith Column. J. Chromatogr. A 1216 (13), 2725–2729. 10.1016/j.chroma.2008.11.010 19041094

[B61] Wörner T. P. Snijder J. Bennett A. Agbandje-McKenna M. Makarov A. A. Heck A. J. R. (2020). Resolving Heterogeneous Macromolecular Assemblies by Orbitrap-Based Single-Particle Charge Detection Mass Spectrometry. Nat. Methods 17 (4), 395–398. 10.1038/s41592-020-0770-7 32152501

[B62] Wright J. F. (2014). AAV Empty Capsids: for Better or for Worse? Mol. Ther. 22 (1), 1–2. 10.1038/mt.2013.268 24384906 PMC3978789

[B63] Wright J. F. (2008). Manufacturing and Characterizing AAV-Based Vectors for Use in Clinical Studies. Gene Ther. 15 (11), 840–848. 10.1038/gt.2008.65 18418418

[B64] Wright J. (2014). Product-Related Impurities in Clinical-Grade Recombinant AAV Vectors: Characterization and Risk Assessment. Biomedicines 2 (1), 80–97. 10.3390/biomedicines2010080 28548061 PMC5423478

[B65] Xiang Z. Gao G. Reyes-Sandoval A. Cohen C. J. Li Y. Bergelson J. M. (2002). Novel, Chimpanzee Serotype 68-based Adenoviral Vaccine Carrier for Induction of Antibodies to a Transgene Product. J. Virol. 76 (6), 2667–2675. 10.1128/jvi.76.6.2667-2675.2002 11861833 PMC135983

[B66] Yang X. Agarwala S. Ravindran S. Vellekamp G. (2008). Determination of Particle Heterogeneity and Stability of Recombinant Adenovirus by Analytical Ultracentrifugation in CsCl Gradients. J. Pharm. Sci. 97 (2), 746–763. 10.1002/jps.21008 17593554

[B67] Zhang C. Chi Y. Zhou D. (2017). Development of Novel Vaccines against Infectious Diseases Based on Chimpanzee Adenoviral Vector. Methods Mol. Biol. 1581, 3–13. 10.1007/978-1-4939-6869-5_1 28374240

[B68] Zhao H. Xu C. Luo X. Wei F. Wang N. Shi H. (2018). Seroprevalence of Neutralizing Antibodies against Human Adenovirus Type-5 and Chimpanzee Adenovirus Type-68 in Cancer Patients. Front. Immunol. 9, 335. 10.3389/fimmu.2018.00335 29563911 PMC5845880

